# Association of increased B7 protein expression by infiltrating immune cells with progression of gastric carcinogenesis

**DOI:** 10.1097/MD.0000000000014663

**Published:** 2019-02-22

**Authors:** Lingchuan Guo, Zhiju Liu, Yun Zhang, Qiuying Quan, Lili Huang, Yunyun Xu, Lei Cao, Xueguang Zhang

**Affiliations:** a Jiangsu Institute of Clinical Immunology, The First Affiliated Hospital of Soochow University; Department of Pathology, The First Affiliated Hospital of Soochow University; b Department of Pathology, The First Affiliated Hospital of Soochow University; c Department of Clinical Laboratory; d Institute of Pediatric Medicine, Children's Hospital of Soochow University; e Jiangsu Institute of Clinical Immunology, The First Affiliated Hospital of Soochow University; f Jiangsu Institute of Clinical Immunology, The First Affiliated Hospital of Soochow University; Jiangsu Key Laboratory of Clinical Immunology, Soochow University; Jiangsu Key Laboratory of Gastrointestinal tumor Immunology, Suzhou, PR China.

**Keywords:** B7 costimulatory molecules, carcinogenesis, gastritis, immunohistochemistry, neoplasms

## Abstract

B7 negative costimulatory molecules are a group of molecules associated with the occurrence, development, and therapy of cancers. Here, we aimed to determine the clinical significance of PD-L1, B7-H3, and B7-H4 and their expression in CD8 and CD68 positive cells at different stages of gastric carcinogenesis.

We detected PD-L1, B7-H3, B7-H4, CD8, and CD68 expression in samples by immunohistochemical staining of 62 chronic superficial gastritis (CSG) samples, 72 chronic atrophic gastritis (CAG) samples, 68 low-grade intraepithelial neoplasia (LIN) samples, 65 high-grade intraepithelial neoplasia (HIN) samples obtained from gastroscopic biopsies and 50 gastric adenocarcinoma (GA) samples obtained from surgical resections. Then we statistically analyzed the expression differences and correlations.

Our results indicated that B7 and CD68 expression on infiltrating immune cells was associated with disease progression. However, infiltration of CD8+ cells decreased with disease progression. B7-H3 expression was markedly enhanced at neoplasia and GA stages. B7-H3 in tumor cells was negatively correlated with CD8-expressing cells. Conversely, B7-H3 expression in tumor-infiltrating immune cells was positively correlated with CD68-expressing cells. B7-H4 expression was found in the cell membrane at the stages of gastritis and low-grade neoplasia and was gradually expressed in the cytoplasm at high-grade neoplasia and GA stages. High B7-H4 expression in infiltrating immune cells was also significantly associated with lower CD8-positive and higher CD68-positive cell densities.

Increased B7 protein expression by infiltrating immune cells was associated with disease progression, and specifically, the level of B7-H3 expression and localization of B7-H4 expression differed significantly among different stages of gastric carcinogenesis.

## Abstract 2

Supplemental Digital Content is available in the text

## 1 Introduction

Gastric cancer is one of the deadliest malignancies. The incidence and mortality of gastric cancer are ranked as the third and fourth of malignant tumors in China.^[1]^ Gastric adenocarcinoma (GA) arises through multiple steps originating with chronic superficial gastritis (CSG), progressing through stages of chronic atrophic gastritis (CAG), then intraepithelial neoplasia and finally into invasive carcinoma.^[2]^ A variety of genes, cytokines, and proteins are implicated in these complex processes. Therefore, studies on these characters will be of great significance for the early diagnosis of gastric precancerous lesions.^[3,4]^ At present, gastroscopy is a necessary diagnostic tool for the detection and treatment of gastrointestinal diseases. It also provides an important screening tool for detecting gastrointestinal malignancies through biopsies.^[5]^ A variety of drugs are used in the clinical treatment of gastric cancer, and inhibition of tumor cell DNA replication is an effective strategy for anti-cancer therapy. Multiple studies indicate that mechanisms affecting DNA replication may play an important role in the development of anticancer drugs and provide new ideas for the treatment of tumors.^[6–10]^

In recent years, immunotherapy has become a major research focus. A series of molecules called B7 family negative costimulatory molecules that mediate cancer immune-regulation such as PD-L1 (B7-H1)/PD-1, B7-H3, and B7-H4, are abnormally expressed in various tumor tissues.^[11]^ These molecules are important components of the tumor microenvironment, and they are involved in the process of immune-escape and are related to tumor clinical pathology and prognosis.^[12,13]^ In this study, we examined the expression levels of PD-L1, B7-H3, and B7-H4 proteins as well as CD8 and CD68 on infiltrating immune cells in CSG, CAG, low-grade intraepithelial neoplasia (LIN), high-grade intraepithelial neoplasia (HIN), and GA. We also studied the expression of negative costimulatory molecules in tumor or immune cells and evaluated the relationship between negative costimulatory molecule expression and immune cell infiltration at the different stages of gastric carcinogenesis.

## 2 Materials and methods

### 2.1 Patients and specimens

This study population comprised 317 patients including 184 males and 133 females, range 22 to 84 years old, (average age 58.6 ± 13.2 years). These patients included 62 cases of CSG, 72 cases of CAG, 68 cases of LIN, 65 cases of HIN and 50 cases of GA diagnosed by 2 qualified pathologists between 2014 and 2017 in the Department of Pathology, the First Affiliated Hospital, Soochow University, Suzhou, China (Table 1). All samples except GA were acquired by gastroscopic biopsy. GA samples were acquired by surgical resection. Biopsy and surgical resection specimens were collected from the antrum of the stomach. All samples were fixed in neutral buffered formalin and embedded in paraffin. This study was approved by the Institutional Review Board of the First Affiliated Hospital of Soochow University (approval number: 2018121).

**Table 1:**
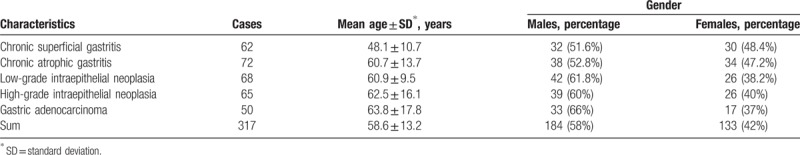
Age and gender of cases.

### 2.2 Immunohistochemical staining

Immunohistochemical staining was performed using the 2-step EnVision method (Dako, Glostrup, Denmark). Paraffin-embedded tissues were cut into 4-μm serial sections, transferred onto adhesive slides and dried at 65°C for 2 hours. The sections were deparaffinized with xylene and rehydrated through graded alcohols. Endogenous peroxidase activity was blocked with 0.3% hydrogen peroxide solution for 30 minutes at room temperature and antigen retrieval was performed by boiling the slides at 110°C for 3 minutes in citrate buffer (pH 6.0). After washing 3 times with PBS for 5 minutes each time, the sections were blocked with 10% normal horse serum followed by incubation with rabbit anti-human PD-L1 monoclonal (1:200; clone number E1L3N; CST), goat anti-human B7-H3 polyclonal (10 μg/mL; R&D systems), rabbit anti-human B7-H4 monoclonal (1:400; clone number EP1165; Abcam, Cambridge, MA), mouse anti-human CD8 monoclonal (clone C8/144B; DAKO), mouse anti-human CD68 monoclonal (clone KP1; DAKO) antibodies at 4°C overnight. Then the slides were washed with PBS for 3 times, followed by incubation with horseradish peroxidase-labeled secondary antibody for 30 minutes. Samples were washed again twice with PBS for 5 minutes each, followed by immunodetection using the Dako EnVision detection system. The slides were counterstained with Mayer's hematoxylin, dehydrated in graded alcohol, and mounted with a neutral resin. The negative control was performed by replacing the primary antibody with mouse or rabbit or goat IgGs. Human tonsil tissue was used as the positive control.

### 2.3 Assessment of tissue staining

All immunohistochemical studies were independently and blindly reviewed by 2 pathologists. All slides were scanned with a Dmetrix image system. Staining was evaluated in columnar epithelial or tumor cells or immune cells. The percentage of positively stained cells was an average after counting the intensely stained and the total number of cells from all fields. Infiltrating immune cells expressing CD8 or CD68 were scored based on the density of the immune cell infiltrate as follows: no CD8 or CD68-positive immune cells: 0; 1–10 CD8 or CD68-positive immune cells per high power field: 1; 11–20 CD8 or CD68-positive immune cells per high power field: 2; 21–30 CD8 or CD68-positive immune cells per high power field: 3, an more than 30 CD8 or CD68-positive immune cells per high power field: 4. The percentage of infiltrating CD8- or CD68-positive immune cells was scored separately in 5% increments. The percentages of columnar epithelial or tumor cells or immune cells that stained positively for PD-L1 or B7-H3 or B7-H4 were quantified in 5% increments. In addition, the intensity of staining was scored from 0 (negative) to 1 (weak) to 2 (moderate) to 3 (intense). If <5% of cells stained positively for PD-L1, B7-H3, or B7-H4, the staining result was considered negative.

### 2.4 Statistical analysis

Statistical analysis was performed using SPSS (v19.0; IBM Corporation, Armonk, NY). Statistical tests were 2-sided, and *P* <.05 was considered statistically significant. Associations between categorical variables were assessed using the Pearson Chi-square test or Fisher exact test, accordingly. Box-and-whisker plots were generated as a non-parametric representation of the distribution of a scale variable in a group; the sides of the box indicate the 1st and 3rd quartiles of the population distribution with a vertical bar indicating the median. The Kruskal–Wallis test was used to identify statistical differences among the 5 groups.

## 3 Results

### 3.1 Expression of PD-L1 protein in the different stages of gastric carcinogenesis

**Figure 1:**
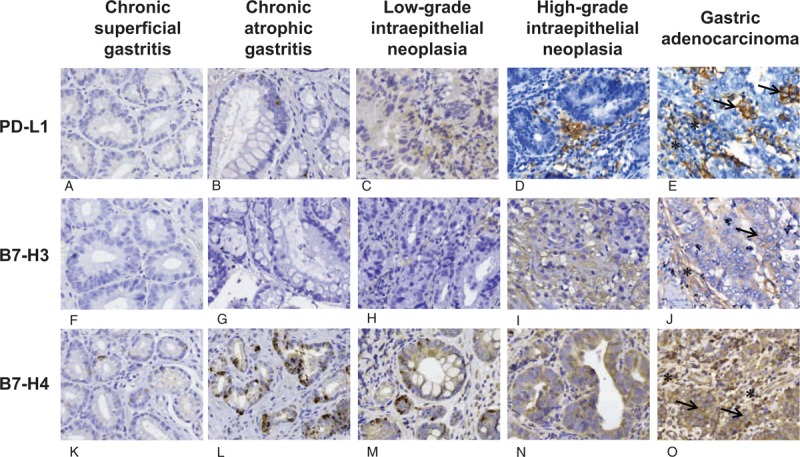
Immunohistochemical characteristics of PD-L1, B7-H3 and B7-H4 expression in superficial gastritis (a,f,k); atrophic gastritis (b, g, l); low-grade intraepithelial neoplasia (c, h, m); high-grade intraepithelial neoplasia (d, i, n); and gastric adenocarcinoma (e, j, o). Arrows point to tumor cells expressing PD-L1, B7-H3, or B7-H4, and stars point to tumor-infiltrating immune cells expressing PD-L1, B7-H3, or B7-H4. Magnification: 40×.

**Figure 2:**
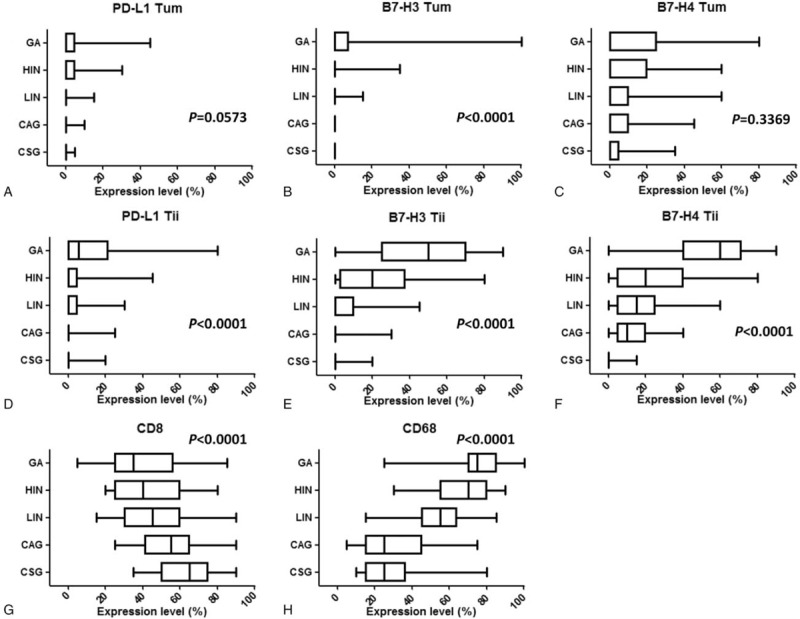
Distributions of PD-L1, B7-H3, and B7-H4 expression and CD8 and CD68 density in the different stages of gastric carcinogenesis. Box-and-whiskers plot of the distributions of negative costimulatory molecules and immune cell markers according to the different stages of gastric carcinogenesis: CAG = chronic atrophic gastritis, CSG = chronic superficial gastritis, GA = gastric adenocarcinoma, HIN = high-grade intraepithelial neoplasia, LIN = low-grade intraepithelial neoplasia. a,b,c percentages of tumor cells expressing PD-L1, B7-H3, and B7-H4 in the 5 stages; d,e,f percentages of tumor-infiltrating immune cells expressing PD-L1, B7-H3, and B7-H4 in the 5 stages; g,h percentages of tumor-infiltrating immune cells expressing CD8 and CD68 in the 5 stages.

By immunohistochemistry, we analyzed 62 cases of CSG, 72 cases of CAG, 68 cases of LIN, 65 cases of HIN and 50 cases of GA. As shown in Figure 1a–e, PD-L1 protein was primarily expressed in the tumor/parenchymal cell and immune cell membrane and/or cytoplasm based on DAB staining. In tumor/parenchymal cells, PD-L1 was expressed with low levels in all stages (Fig. 2a). In immune cells, PD-L1 protein was hardly detected in CSG and CAG, but weakly expressed in LIN and HIN and moderately expressed in GA (Fig. 2d). Interestingly, in intraepithelial neoplasia and GA, PD-L1 expression was mostly localized at cancer nests edge of stroma adjacent to cancer cells, suggesting that PD-L1 expression may be implicated in infiltrating immune cells and promotes tumor immune escape (Fig. 1c–e). The percentages of PD-L1-positive cases among the CSG, CAG, LIN, HIN, and GA specimens were 8.1% (5/62), 18.1% (13/72), 32.4% (22/68), 36.9% (24/65), and 42% (21/50) (Table 2), respectively.

**Table 2:**
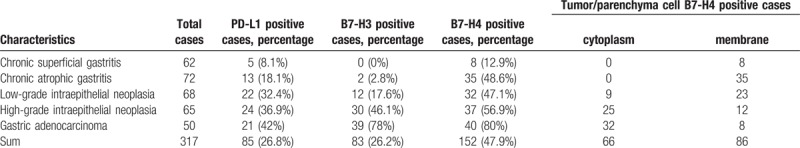
PD-L1, B7-H3 and B7-H4 expression in the multistage tissue of gastric carcinogenesis.

### 3.2 Expression of B7-H3 protein in the different stages of gastric carcinogenesis

B7-H3 protein was primarily expressed in the tumor/parenchymal cell and immune cell membrane and/or cytoplasm (Fig. 1f–j). In tumor/parenchymal cells, B7-H3 was mainly expressed in the gastric cancer stage and hardly expressed in the other stages (Fig. 2b). In infiltrating immune cells, B7-H3 expression showed a significant increasing trend with disease progression: almost no B7-H3 expression was detected in the inflammatory phase, but B7-H3 expression began and then gradually increased during the neoplastic stage, with a more significant increase in the GA stage than in the neoplastic stage (Fig. 2e). The percentages of B7-H3-positive cases among the CSG, CAG, LIN, HIN, and GA specimens were 0% (0/62), 2.8% (2/72), 17.6% (12/68), 46.1% (30/65), and 78% (39/50) (Table 2), respectively.

### 3.3 Expression of B7-H4 in the different stages of gastric carcinogenesis

In CSG, a small amount of B7-H4-positive cells was sporadically located in the mucosa of sinus ventriculi. In CAG, B7-H4 was expressed in atrophic gastric epithelial cells. In this phase, infiltrating immune cells also expressed B7-H4. In LIN, B7-H4 was moderately and sporadically expressed in neoplastic cells. In HIN, B7-H4 expression was strongly diffuse in some of the infiltrating immune cells compared with that in low-grade neoplasia. Importantly, B7-H4 was diffuse in almost all tumor cells and a large amount of infiltrating immune cells in GA (Figs. 1k-o, 2c,f). During the progression of gastric cancer, there was no significant change in B7-H4 expression by tumor/parenchymal cells (Fig. 2c), but the expression of B7-H4 in infiltrating immune cells changed significantly with a gradually increasing trend (Fig. 2f). The percentages of B7-H4-positive cases among the CSG, CAG, low-grade intraepithelial neoplasia, HIN, and GA specimens were 12.9% (8/62), 48.6% (35/72), 47.1% (32/68), 56.9% (37/65), and 80% (40/50) (Table 2), respectively. Of note, B7-H4 was mainly expressed on cell membranes in CSG and CAG, whereas in intraepithelial neoplasia and GA, B7-H4 expression was mostly localized in the cytoplasm of tumor cells (Table 2). In all stages of carcinogenesis, the scores for B7-H4 expression were markedly higher than those for PD-L1 and B7-H3 expression (Fig. 2c and f).

### 3.4 Changes in the expression of negative costimulatory molecules and immune molecules in the different stages of GA

Interestingly, the expression levels of CD8 and CD68 were reversed during the progressive stages of gastric cancer. CD8 expression gradually decreased with disease progression (Fig. 2g), whereas CD68 expression gradually increased (Fig. 2h).

We additionally quantified the percentages of tumor cells expressing PD-L1 (PD-L1 expressing in tumor cells [Tum]), B7-H3 (B7-H3 Tum), and B7-H4 (B7-H3 Tum); the percentages of tumor-infiltrating immune cells expressing PD-L1 (PD-L1 expressing in tumor-infiltrating immune cells [Tii]), B7-H3 (B7-H3 Tii), and B7-H4 (B7-H4 Tii); and the percentages of peritumoral and tumor stroma infiltrated by CD8-positive lymphocytes (CD8) or CD68-positive macrophages (CD68). The results of comparative analyses between CSG and CAG, gastritis and neoplasia, and GA and neoplasia are shown in Table 3. B7-H4 Tii and CD8 expression differed significantly in CSG versus CAG, whereas PD-L1 Tii, B7-H3 Tum, B7-H3 Tii, B7-H4 Tii, CD8, and CD68 expression differed significantly between gastritis versus neoplasia. Likely, in the comparison between GA and neoplasia, PD-L1 Tii, B7-H3 Tum, B7-H3 Tii, B7-H4 Tii, and CD68 expression levels were notably different.

**Table 3:**
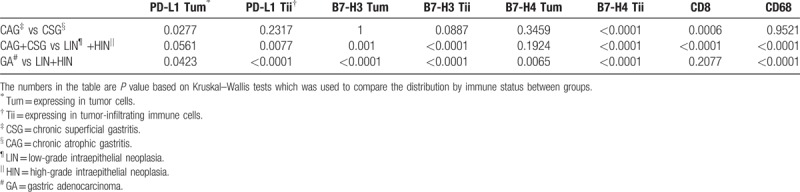
Comparison of quantitative expression of tumor immunology molecules by different stages.

### 3.5 Relationship between negative costimulatory molecule expression and density of CD8- or CD68-positive infiltrating cells

**Figure 3:**
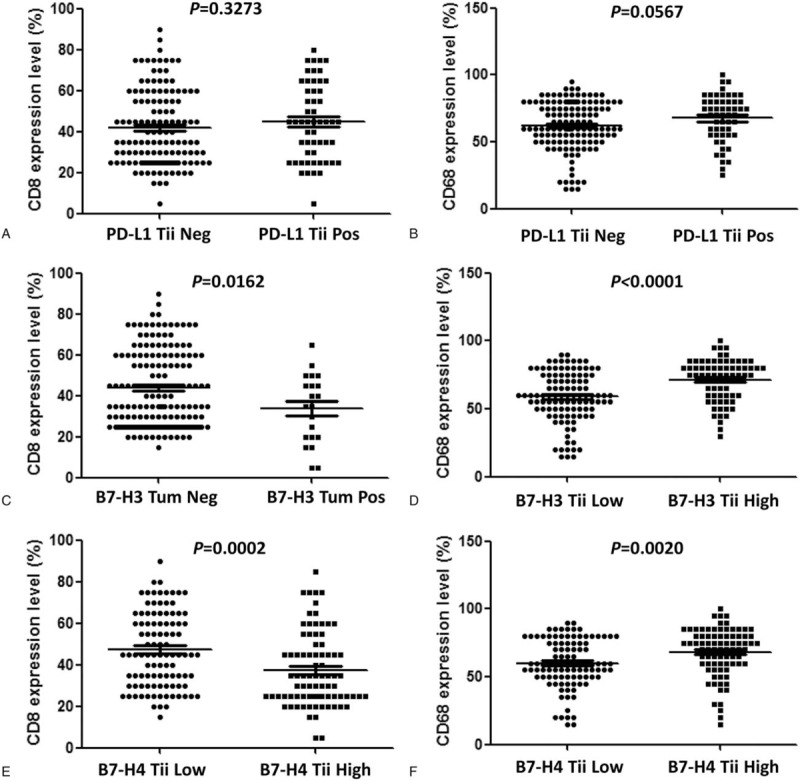
Associations of negative costimulatory molecule expression and the percentage of infiltrating cells expressing CD8 and CD68 in the neoplasia and gastric adenocarcinoma stages. a PD-L1 expression in tumor-infiltrating immune cells and CD8 density; b PD-L1 expression in tumor-infiltrating immune cells and CD68 density; c B7-H3 expression in tumor cells and CD8 density; d B7-H3 expression in tumor-infiltrating immune cells and CD68 density; e B7-H4 expression in tumor-infiltrating immune cells and CD8 density; f B7-H4 expression in tumor-infiltrating immune cells and CD68 density.

We then examined the relationship between the expression of these negative costimulatory molecules and the densities of infiltrating CD8- and CD68-positive cells in the GA and neoplasia phases (Fig. 3). The densities of CD8-positive cells and CD68-positive cells did not significantly correlated with PD-L1 Tii expression (Fig. 3a and b CD8 *P* = .3273; CD68 *P* = .0567). However, the density of CD8-positive cells in B7-H3 Tum positive samples was significantly lower than that in B7-H3 Tum-negative samples (Fig. 3c *P* = .0162; Supplementary Figure. 1). The density of CD68-positive cells in samples with high B7-H3 Tii expression was significantly higher than that in samples with low B7-H3 Tii expression (Fig. 3d *P* <.0001; Supplementary Figure. 2). High B7-H4 expression by infiltrating immune cells (Tii) was also significantly associated with a lower density of CD8-positive cells and a higher density of CD68-positive cells (Fig. 3e,f CD8 *P* = .0002; CD68 *P* = .0020). In the gastritis phases, the density of CD68-positive cells in samples with high B7-H4 Tum expression was significantly higher than that in samples with low B7-H4 Tum expression (Supplementary Figure. 3, *P* = .0216). There were no significant correlations between B7-H4 Tum and CD8 expression (*P* = .9077), B7-H4 Tii and CD8 expression (*P* = .0554), or B7-H4 Tii and CD68 expression (*P* = .4292). The detected expression levels of PD-L1 and B7-H3 were too low to evaluate.

## 4 Discussion

Although the etiology of GA is not completely clear, a large amount of data indicates that gastric cancer is closely related to *Helicobacter pylori* infection, environmental factors, eating habits, and genetic factors.^[14]^ Studies have confirmed that the infection rate of *H pylori* has a significant correlation with the mortality of gastric cancer. As *H pylori* is involved in the development of gastric cancer, inhibition of *H pylori* is an effective means of preventing gastric cancer.^[15]^ The pathogenesis of *H pylori* leading to the development of gastric cancer is multifaceted, including involvement of DNA damage, epigenetic modifications, and induction of cancer cell invasion and metastasis. *H pylori* may also affect the biological function of cancer stem cells and induction of cell autophagy.^[16,17]^ Immune evasion strategies and persistence of *H pylori* are also important directions of research.^[18]^

In this study, we investigated the tissue expression patterns of PD-L1, B7-H3, and B7-H4 in the different stages of gastric carcinogenesis. PD-L1 protein is abnormally expressed on the surface of many tumor cells, including gastric cancer.^[19–21]^ Its expression in tumor cells is closely related to the occurrence and development of tumors and prognosis of patients.^[22]^ However, there have been few investigations of the role of PD-L1 in various stages of tumor progression, especially in the early stage. Previous studies also demonstrated increased PD-L1 expression in human gastric epithelial cells in *H pylori* infection.^[23,24]^ Although we found PD-L1 expression was increased from gastritis to neoplasm, its expression level was not high enough to be used as an early diagnostic indicator of gastric cancer. From our results, the expression of PD-L1 in immune cells differed significantly from that during the neoplasia and gastritis stages as well as that during the adenocarcinoma and neoplasia stages, indicating that PD-L1 expression by immune cells may play an important function in the immune microenvironment. This result was consistent with previous reports that the level of PD-L1 expression in immune cells is related to patient prognosis^[25]^ or the therapeutic effect of PD-L1/PD-1 monoclone antibody (mAb),^[26]^ but not PD-L1 expression by tumor cells. In addition, our results showed that the expression of PD-L1 Tii was not significantly correlated with the infiltration of CD68-expressing cells during the neoplasia and adenocarcinoma stage (*P* = .0567), whereas Kazuto Harada et al^[27]^ considered that PD-L1 expression was positively correlated with the infiltration of CD68-positive cells. In tumor tissues, PD-L1 inhibits T cell killing mainly via binding to PD-1 on killer T cells. Recent studies have shown that T cell-dendritic cell (DC) crosstalk is required for antibody therapy with PD-1, and DCs may play a more important role in mediating PD-L1-PD-1 signaling.^[28]^ On the other hand, the transforming growth factor-beta (TGF-β) pathway may also affect treatment with PD-L1, and research has shown that TGF-β attenuates the tumor response to PD-L1 blockade by contributing to the exclusion of T cells.^[29]^ As TGF-β is mostly secreted by fibroblasts, this may suggest that fibroblasts are associated with the PD-L1-PD-1 axis. In our study, we found that B7-H3 expression was associated with CD68 expression in the progression of gastric carcinogenesis, but there was no correlation between PD-L1 and CD68 expression, which may also imply that CD68 may interact with other costimulatory molecules. Considering the above biological hypothesis and that our statistical scope included the neoplasia stage and only 50 cases GA, our results showing no significant relation between PD-L1 and CD68 expression seem acceptable.

Unlike PD-L1, B7-H3 showed a clear change in expression during the different stages of gastric carcinogenesis. In recent years, B7-H3 has been extensively studied as a negative modulator in tumor immunity.^[30]^ B7-H3 protein is expressed in many tumor tissues, such as lung cancer, gastric cancer, kidney cancer, prostate cancer, and neuroblastoma.^[31,32]^ Studies on B7-H3 expression in gastric cancer have indicated that ectopic expression of B7-H3 is closely related to tumor progression and poor prognosis.^[33,34]^ B7-H3 overexpression has been linked to poor prognosis in human patients and to the invasive and metastatic potential of tumors in vitro models. Moreover, recent evidence has shown that B7-H3 influences cancer progression beyond the immune regulatory roles.^[30,35]^ In our comparison of CSG and CAG, no significant difference in B7-H3 expression was observed in either tumor cells or immune cells. However, we found that in both tumor cells and immune cells, B7-H3 expression was significant altered during the gastritis-neoplasia transition, indicating that B7-H3 might serve as an early diagnostic indicator of gastric cancer. We found that few studies have reported the relationship of B7-H3 expression in gastritis and neoplasia. Our comparison between the cancer and neoplastic stages also showed an obvious change in B7-H3 expression, with a significant increase in B7-H3 expression during the gastric tumor stage. These findings suggest that B7-H3 is closely related to cancer progression. In liver cancer, the finding that the soluble B7-H3 level in cirrhotic patients with hepatocellular carcinoma is higher than that in cirrhotic and healthy patients was consistent with our findings.^[36]^

In addition, we discovered whether B7-H3 was expressed in parenchymal or stromal cells, it was significantly associated with the infiltration of CD8- and CD68-positive cells at various stages. At the neoplasia and tumor stages, B7-H3-positive tumor cells and CD8-positive T cells showed a negative correlation, suggesting that B7-H3 has different characteristics compared to PD-L1. It is possible that B7-H3 independently regulates tumorigenesis through some signaling mechanisms in tumor cells, which is consistent with our other study, in which B7-H3 was found to control the stemness characteristics of colorectal cancer (our unpublished data). Considering B7-H3 can induce tumor necrosis factor-alpha (TNF-α) expression in monocyte-macrophage cells,^[37]^ we assume that B7-H3 is likely to promote the production of some cytokines to influence tumor progression through interaction with CD68-positive cells.

The expression of B7-H4 protein was also hardly detected in normal human tissues, but its expression was significantly increased in tumors including gastric cancer.^[38]^ Recent studies showed that aberrant B7-H4 expression corresponds to a poor prognosis in multiple tumors,^[39,40]^ suggesting that B7-H4 may be implicated in cancer development and progression. In the present study, we found differences in the level and location of B7-H4 expression during the different stages of disease progression: scattered expression and cell membrane localization emerged in the gastritis and early stages of neoplasia, while extensive expression and cytoplasm location were dominant in the late stages of neoplasia and the tumor stage. We have previously found that B7-H4 promotes tumor progression and cell proliferation by translocating into the nucleus in renal cell carcinoma tissues.^[41]^ We hypothesized that changes in the subcellular location of B7-H4 may be closely related to its functions. Therefore, research of the mechanism of B7-H4 localization is of great importance for understanding the functions of B7-H4 in tumors.

Similar to B7-H3, B7-H4 was also highly expressed in infiltrating immune cells during the cancer and neoplasia stages. However, unlike B7-H3, B7-H4 expression occurred in the gastritis stage, and immune cells expressing B7-H4 gradually increased as the disease progressed. A study showed that B7-H4 is involved in the pathological changes of rheumatoid synovium in rheumatoid arthritis progression and expressed on CD19(+) B cells and CD14(+) monocytes, but not on CD3(+) T cells.^[42]^ According to our results, immune cell expression of B7-H4 seemed to be more consistent with the progression of the disease than tumor cell expression of B7-H4. With progression through the gastritis-neoplasia-tumor stages, the expression of B7-H4 in immune cells gradually increased. B7-H4 expressed by immune cells may inhibit the infiltration of T cells, resulting in the loss of immune surveillance and further leading to gastric carcinogenesis. On the other hand, our study found that the expression of B7-H4 in immune cells was positively correlated with the infiltration of CD68-positive cells during gastric cancer progression, but negatively related with the infiltration of CD8-positive cells. Similarly, Li et al reported that increased B7-H4 expression on myeloid cells from human hepatocellular carcinoma correlated with CD8+ T-cell dysfunction. The percentage of Granzyme B+Perforin+CD8+ tumor-infiltrating lymphocytes (TILs) negatively correlates with the percentage of B7-H4+ cells in tumors.^[43]^

CD8 and CD68 have been reported to be associated with the development and prognosis of gastric cancer. Some reports indicated that intratumoral CD68 and CD8 infiltration is positively correlated with the prognosis of patients.^[44,45]^ Others reported that infiltrating CD68-positive macrophages are associated with poor prognosis.^[46,47]^ Our results showed that CD8 expression gradually decreased with the progression of GA, whereas CD68 expression gradually increased. Moreover, CD8 expression differed significantly in the CSG versus CAG stages as well as in the inflammatory versus neoplastic stages, while CD68 expression differed significantly in the inflammatory versus neoplastic stages and the tumor versus neoplastic stages, suggesting that CD8 functions in the early stages of disease progression, whereas CD68 plays a more important role after the neoplastic phase.

To our knowledge, this is the first report to elucidate the expression of PD-L1, B7-H3, and B7-H4 in infiltrating immune cells in multiple steps of gastric carcinogenesis. The results of this study clearly indicate that expression of the PD-L1, B7-H3, and B7-H4 molecules follow different patterns in different stages of gastric carcinogenesis. The expression of these 3 costimulatory molecules in immune cells seems to be more closely related to the progression of the disease. No significant correlation was found between PD-L1 expression and the infiltration of immune cells. Compared with PD-L1 expression in tumor/parenchymal cells, the expression of B7-H3 in tumor/parenchymal cells was more strongly correlated with CD8-positive and CD68-positive cell infiltration, while B7-H4 expression was associated with the infiltration of immune cells only in stromal cells, and the localization of B7-H4 in tumor/parenchymal cells was more significantly associated with tumor progression.

## Author contributions

**Conceptualization:** Lei Cao.

**Data curation:** Lingchuan Guo, Zhiju Liu, Yun Zhang, Lei Cao.

**Formal analysis:** Lei Cao.

**Funding acquisition:** Lili Huang, Yunyun Xu, Lei Cao, Xueguang Zhang.

**Investigation:** Zhiju Liu, Lili Huang.

**Methodology:** Zhiju Liu, Yun Zhang, Qiuying Quan, Yunyun Xu.

**Supervision:** Xueguang Zhang.

**Writing – original draft:** Lingchuan Guo.

**Writing – review & editing:** Lei Cao.

## Supplementary Material

Supplemental Digital Content
