# Supplementary figures and images for: Association of increased B7 protein expression by infiltrating immune cells with progression of gastric carcinogenesis
Source: Medicine (Baltimore). 2019 Feb 22;98(8):e14663. doi: 10.1097/MD.0000000000014663 (PMC6407991; doi:10.1097/MD.0000000000014663)

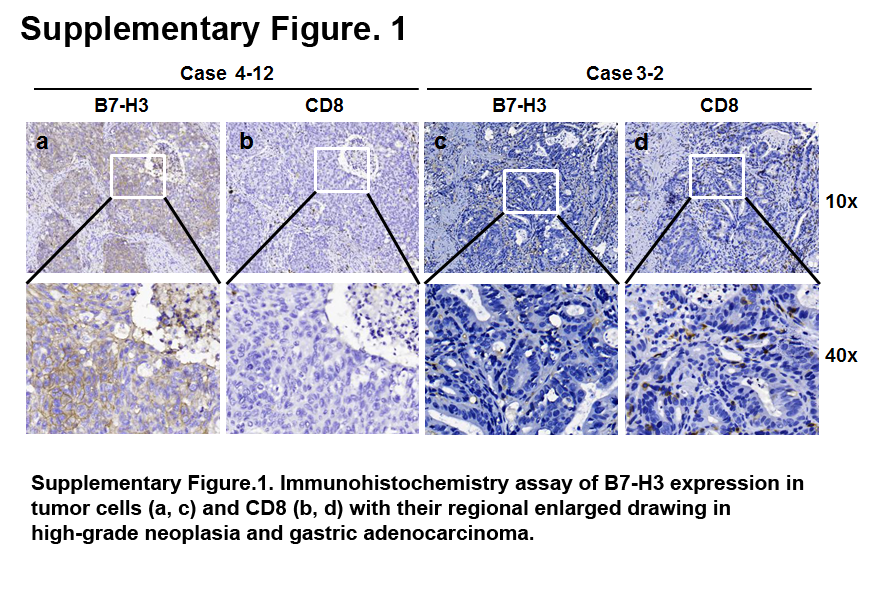


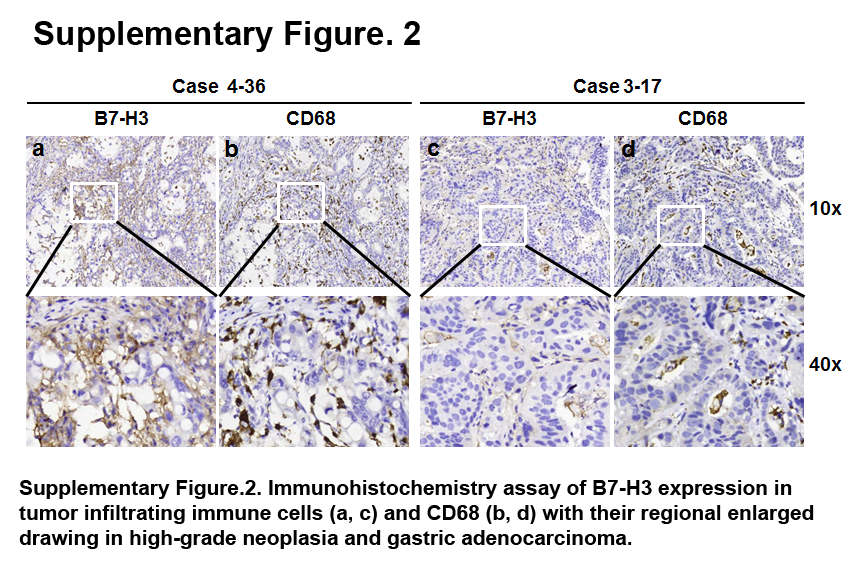


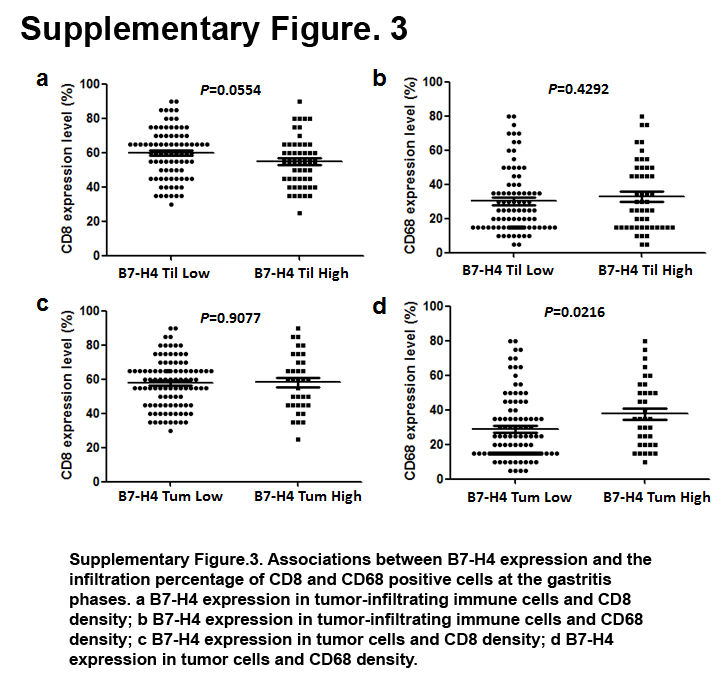

Supplement: Supplemental Digital Content [file medi-98-e14663-s001.doc]
